# Predictive value of ELWI combined with sRAGE/esRAGE levels in the prognosis of critically ill patients with acute respiratory distress syndrome

**DOI:** 10.1038/s41598-023-42798-4

**Published:** 2023-09-19

**Authors:** Chengliang Zhang, Dekun Yin, Xi Zhu, Wenshuo Zhou, Zhihua Xu, Liuping Wu, Weili Gu

**Affiliations:** 1 Department of Intensive Care Medicine, The Second Affiliated Hospital of Nantong University, 6# North Road, Child Lane, Chongchuan District, Nantong, 226001 Jiangsu China; 2 Department of Anesthesiology, Funing People’s Hospital of Jiangsu, Yancheng, 224400 Jiangsu Province China; 3 Grade 21, Clinical Medicine, Nantong University Medical School, Nantong, 226001 Jiangsu China; 4 Department of Anesthesiology, Affiliated Hospital of Nantong University, Nantong, 226001 China

**Keywords:** Biomarkers, Medical research, Molecular medicine, Risk factors

## Abstract

Acute respiratory distress syndrome (ARDS) is a life-threatening condition. Accurate judgement of the disease progression is essential for controlling the condition in ARDS patients. We investigated whether changes in the level of serum sRAGE/esRAGE could predict the 28-day mortality of ICU patients with ARDS. A total of 83 ARDS patients in the ICU of the Second Affiliated Hospital of Nantong University from January 2021 to June 2022 were consecutively enrolled in this study. Demographic data, primary diagnosis and comorbidities were obtained. Multiple scoring systems, real-time monitoring systems, and biological indicators were determined within 6 h of admission. The clinical parameters for survival status of the ARDS patients were identified by multivariate logistic regression. Receiver operating characteristic (ROC) curve analysis was employed to verify the accuracy of the prognosis of the related parameters. The admission level of sRAGE was significantly higher in the nonsurvival group than in the survival group (*p* < 0.05), whereas the serum esRAGE level showed the opposite trend. Multivariate logistic regression analysis showed that sRAGE (AUC 0.673, *p* < 0.05), esRAGE (AUC 0.704, *p* < 0.05), and ELWI (extravascular lung water index) (AUC 0.717, *p* < 0.05) were independent risk factors for the prognosis of ARDS. Model B (ELWI + esRAGE) could not be built as a valid linear regression model (ELWI, *p* = 0.079 > 0.05). Model C (esRAGE + sRAGE) was proven to have no significance because it had a predictive value similar to that of the serum levels of esRAGE (Z = 0.993, *p* = 0.351) or sRAGE (Z = 1.116, *p* = 0.265) alone. Subsequently, Model D (sRAGE + esRAGE + ELWI) showed the best 28-day mortality predictive value with a cut-off value of 0.426 (AUC 0.841; *p* < 0.001), and Model A (sRAGE + ELWI) had a cut-off value of 0.401 (AUC 0.820; *p* < 0.001), followed by sRAGE (AUC 0.704, *p* = 0.004), esRAGE (AUC 0.717, *p* = 0.002), and ELWI (AUC 0.637, *p* = 0.028). In addition, there was no statistically significant difference between Model A and Model D (Z = 0.966, *p* = 0.334). The admission level of sRAGE was higher in the nonsurvival group, while the serum esRAGE level showed the opposite trend. Model A and Model D could be used as reliable combined prediction models for predicting the 28-day mortality of ARDS patients.

## Introduction

Acute respiratory distress syndrome (ARDS), which is characterized by acute hypoxemia, respiratory distress, and pulmonary exudative lesions, is a highly heterogeneous and complicated critical illness secondary to pneumonia, sepsis, trauma, or blood transfusion^1^. Globally, ARDS accounts for 10% of intensive care unit admissions and approximately 35–46% of hospital mortality^2^. Survivors of ARDS typically suffer long-term sequelae, including physical, cognitive, and psychological diseases, and the 3-month long-term mortality ranges from 38 to 41.2%^3^, bringing a great economic burden to families and society. With the lack of effective drug interventions, the accurate judgement of disease status and effective clinical management are critically important for patients with ARDS.

Currently, there are several scoring systems for the early identification of patients at risk of developing ARDS and the prediction of outcomes in ARDS patients, including the oxygenation ratio PaO_2_/FiO_2_, extravascular lung water index (ELWI), Murray lung injury score (MLIS), and acute physiology and chronic health evaluation (APACHE II)^4–7^. Despite traditionally being considered purely prognostic indicators, both the oxygenation ratio and the oxygenation index had low sensitivity and specificity in the early diagnosis and lagged behind the actual progression of ARDS^8^. ELWI, as a complementary nonspecific index, was employed for the diagnosis of pulmonary oedema and to predict the progression of acute lung injury. However, pulmonary oedema can develop in multiple diseases, including neurogenic pulmonary oedema and high-altitude pulmonary oedema, in addition to ARDS^5,9,10^. All types of scoring systems, such as the Murray lung injury score and APACHE II, were very useful to predict the risk of mortality and to grade the severity of disease; however, these systems also led to some discrepancies in judgement because of the subjective assessment. Hence, it is of paramount importance to find biological diagnostic indicators with high specificity and sensitivity to solve this conundrum.

The receptor for advanced glycation end products (RAGE) is a cell-surface multiligand member of the immunoglobulin superfamily that carries out multiple biological functions in many disease models, such as diabetic complications, decreased renal function, atherosclerosis, and metabolic syndrome^11,12^. A growing number of studies have revealed that elevated plasma levels of cell surface RAGE (sRAGE) have been detected in an animal lung injury model when measured at baseline, which may reflect the severity of lung epithelial injury^13,14^. RAGE inhibition or RAGE gene knockout was found to significantly attenuate pulmonary capillary permeability and reduce inflammatory reactions in lung ischaemia‒reperfusion injury model mice^15^. Moreover, sRAGE could be considered a quantitative reflective index for alveolar epithelial and endothelial damage induced by the systemic inflammatory response in patients with acute lung injury (ALI)^16^. Despite the possible correlation between sRAGE and lung injury-related diseases, relevant clinical study data are still inadequate in patients with ARDS. On the other hand, in contrast to sRAGE, endogenous secretory RAGE (esRAGE) is a truncated form cleaved from sRAGE that lacks the transmembrane and cytoplasmic portions of the receptor^17,18^. esRAGE can prevent cell-bound sRAGE signalling from binding to its associated ligands by competitive inhibition, serving as a decoy that abolishes cell activation^18,19^. Although many studies have been performed regarding the effects of esRAGE levels on morbidity in associated conditions, including diabetes and preeclampsia, there are few reports regarding the correlation between esRAGE and ARDS in the literature.

In this prospective study, we aimed to evaluate the value of serum sRAGE and esRAGE as biomarkers reflecting disease prognosis and search for a practical prediction model in critically ill patients with ARDS.

## Materials and methods

### Trial registration and ethics

We confirm that the present study was performed according to the guidelines of the Declaration of Helsinki. This prospective study was approved by the Ethical Review Committee of the Second Affiliated Hospital of Nantong University (No: 2020KY026) and registered at www.chictr.org.cn (number: ChiCTR2100041618). Informed consent was obtained from patients or the nearest relative prior to the study.

### Setting and patients

**Figure 1:**
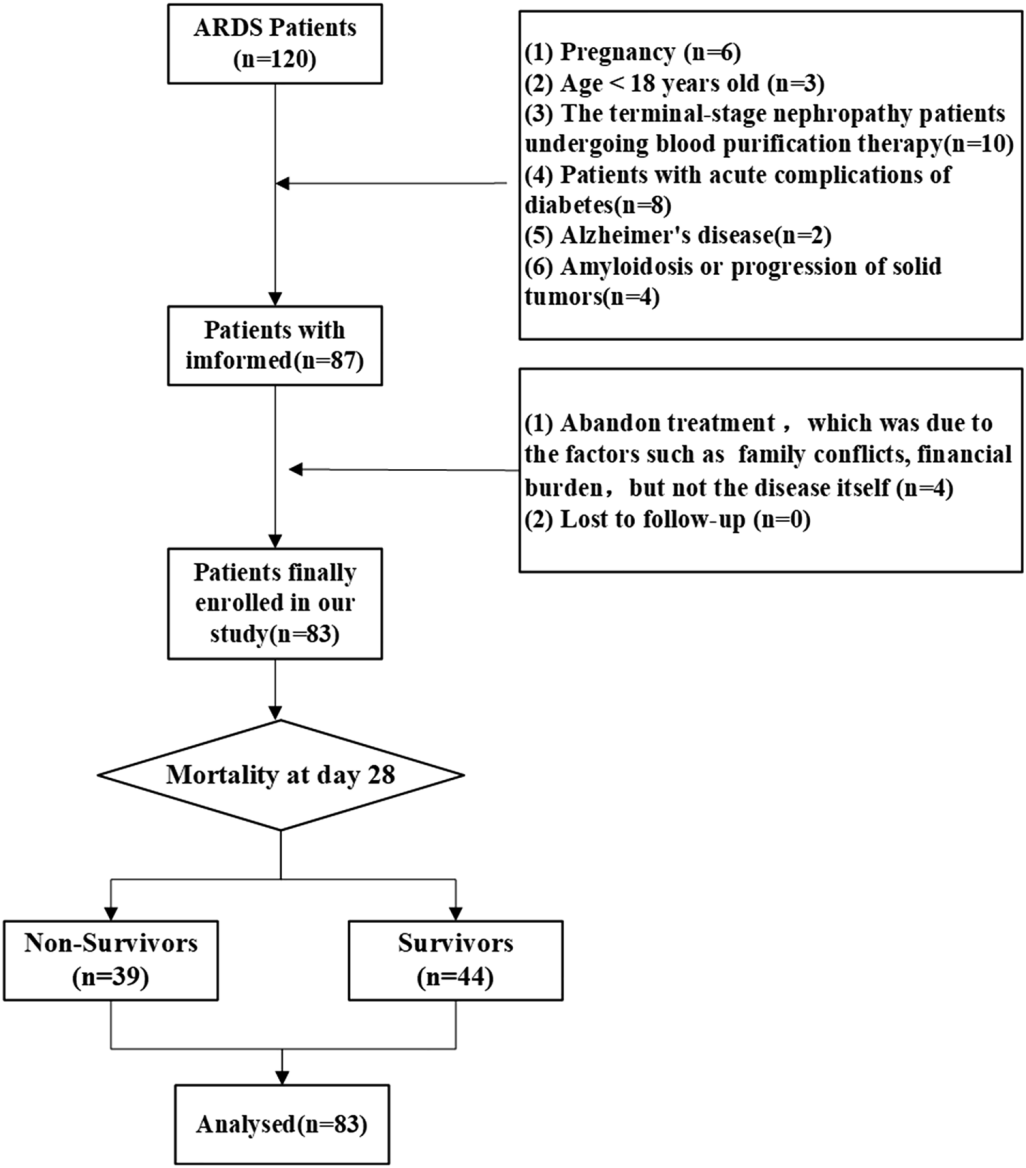
Details of subject enrolment and reason for exclusion from the present study.

In this prospective cohort study, 120 ARDS patients were recruited from the intensive care unit (ICU) of the Second Affiliated Hospital of Nantong University from January 2021 to June 2022. Finally, a total of 83 patients with ARDS were enrolled in the study and further divided into a survival group and a nonsurvival group according to the patients’ outcomes (Fig. 1). All patients with ARDS were diagnosed according to the Berlin definition^20^, and the treatment followed the guidelines for the diagnosis and treatment of acute lung injury/ARDS in the Society of Critical Care Medicine, Chinese Medical Association.

The exclusion criteria of all the patients mentioned above were as follows: (1) pregnancy, (2) age < 18 years old, (3) terminal-stage nephropathy patients undergoing blood purification therapy, (4) patients with acute complications of diabetes, (5) Alzheimer’s disease, (6) amyloidosis or progression of solid tumours, and (7) readmission to the ICU.

### Clinical data collection

Demographic data, including age, sex, temperature, heart rate and mean arterial pressure, were recorded when the patients were admitted to the ICU. Subsequently, all patients were subjected to continuous ECG monitoring, and then the PiCCO (pulse indicator continuous cardiac output) catheter was inserted into the femoral artery, which was used for the measurement of cardiac parameters and pulmonary oedema, such as PVPI (pulmonary vascular permeability index) and ELWI, which could be quantitatively measured through the transpulmonary thermodilution (TPTD) technique. PVPI reflected the alveolar-capillary permeability, and ELWI was used as an index of lung water content and oedema.

Next, all patients underwent standard laboratory investigations and imaging tests that included complete blood counts; blood electrolyte measurements; liver and renal function tests; analysis of electrolytes, blood gases and pH; a chest X-ray; and a CT examination.

For ventilated patients, pulmonary gas exchange and ventilatory parameters, including FiO_2_ (inspiratory fraction of oxygen), PaO_2_ (arterial partial pressure of oxygen), pulmonary compliance, P_peak_ (peak inspiratory pressure), PEEP (positive end-expiratory pressure) and so on, were measured when such patients were admitted to the ICU.

In addition, the clinical parameters were assessed for each patient on admission to the ICU, which mainly involved SOFA scores, APACHE II scores, MLIS scores, and severity of ARDS according to the Berlin definition. The patients were assessed and classified into three severity levels of ARDS according to the Berlin definition. The SOFA (sequential organ failure assessment) score was developed as a tool to quantitatively describe the time course of organ dysfunction^21^. The APACHE II (acute physiology and chronic health evaluation) score is a widely used method to describe the disease severity of patients in the ICU^22^. The MLIS (Murray Lung injury score) was also used to describe the disease severity of ARDS^23^. The main clinical outcome measure was 28-day mortality.

### Sample collection and biomarker measurements

Blood samples were obtained within 6 h of admission to the ICU. The plasma was separated and then stored at − 80 °C until further processing. Previously collected and frozen serum samples were used to determine the levels of sRAGE (R&D Systems, Minneapolis, MN) and esRAGE (B-Bridge International, Sunnyvale, CA) by a commercial sandwich enzyme-linked immunosorbent assay (ELISA) kit. During the ELISAs, all measurements were performed according to the manufacturer’s instructions.

### Sample size

Multivariable analyses, such as multiple regression, logistic regression, and factor analysis show interdependence. No exact predictions are truly feasible. Consequently, rules of thumb are often adopted that the sample size should be 5, 10, or 20 times the number of variables^24^. Based on the abovementioned factors, we found 8 variables, and the sample size of surviving and nonsurviving patients was defined as 80 patients.

### Statistical analysis

Statistical analysis was performed using SPSS version 20.0 and MedCalc 20.0. Normally distributed continuous data were analysed through Student’s t test and are presented as the mean ± standard deviation. Nonnormally distributed continuous data were analysed through the Mann‒Whitney U test and are presented herein as the median (IQR). Categorical variables are presented as frequencies (percentages) and were analysed using a chi-square (χ^2^) test or Fisher’s exact test. Univariate and multivariate logistic regression analyses were performed to identify the clinical parameters for survival status in ARDS patients. Receiver operating characteristic (ROC) curve analysis was employed to verify the accuracy of the prognosis of the related parameters. The accuracy of prognosis was determined using the area under the ROC curve (AUC; 95% confidence interval (CI) and *p* value < 0.05). A value of *p* < 0.05 was considered statistically significant.

## Result

### The association between clinical parameters and survival status in ARDS patients

A total of 120 patients with ARDS were recruited, 83 of whom were divided into two groups, a survivor group and a nonsurvivor group, based on 28-day mortality. The clinical characteristics of the study population were recorded.

There were no significant differences between the two groups in the general clinical characteristics, including age, sex, body mass index, and basic vital signs (Table 1; all *p* > 0.05). There were also no significant differences between the two groups in the primary diagnosis, including trauma, pneumonia, sepsis, cardiovascular disease, pancreatitis, and others (Table 1; all *p* > 0.05). Moreover, there were no significant differences between the two groups in pulmonary compliance, pulmonary vascular permeability index (PVPI), or the parameters of mechanical ventilation, including Ppeak, Ppalt, PEEP, FiO_2_, PaO_2_/FiO_2_, and tidal volume (Table 1; all *p* > 0.05).

**Table 1:** The survival status and clinical parameters in ARDS patients.

Variable	Survival status		*p* value
	Survivors (n = 44)	Non-survivors (n = 39)	
Basic characteristics			
Age (year)	58.2 ± 13.5	57.9 ± 11.7	0.912
Male(male) (n)	28	23	0.821
Body mass index (kg/m^2^)	26.4 ± 2.6	27.1 ± 2.8	0.277
Heart rate (times/min)	90.8 ± 10.3	94.2 ± 14.5	0.213
Breathing rate (times/min)	25.8 ± 2.5	25.3 ± 2.5	0.315
Mean arterial pressure (mmHg)	71.7 (66.9–77.3)	71.8 (64.6–78.0)	0.770
Temperature (℃)	37.4 (36.7–38.2)	38.2 (36.6–38.9)	0.107
Primary diagnosis			0.893
Trauma	9	6	
Pneumonia	10	12	
Sepsis	10	11	
Cardiovascular	7	4	
Pancreatitis	5	4	
Others	3	2	
Clinical routine data			
Ppeak (cm H_2_O)	34.9 ± 1.9	35.8 ± 2.0	0.052
Ppalt (cm H_2_O)	25.0 ± 3.0	25.2 ± 3.0	0.789
Peep (cm H_2_O)	12.0 (10.5–13.0)	13.0 (10.5–14.0)	0.111
FiO_2_	68.7 ± 17.7	67.1 ± 16.5	0.653
PaO_2_/FiO_2_	139.9 (93.5–176.0)	97.6 (84.5–155.9)	0.059
Pulmonary compliance (ml/cmH_2_O)	32.9 ± 7.1	30.1 ± 7.6	0.087
Tidal volume(ml/Kg)	5.8 ± 1.0	6.2 ± 1.0	0.136
Mechanical ventilation (hours)	384 (300–342)	440 (360–505)	0.021*
ICU length of stay (days)	16.0 ± 4.8	18.4 ± 5.5	0.032*
Extravascular lung water index (ELWI)	11.7 ± 2.7	13.0 ± 3.2	0.040*
PVPI	2.61 ± 0.36	2.79 ± 0.54	0.067
Severity of illness scores at ICU admission			
SOFA	12.3 ± 2.6	13.7 ± 3.3	0.037*
APACHE II	18.9 ± 5.8	22.2 ± 6.3	0.014*
Murray lung injury score (MLIS)	1.9 ± 0.8	2.5 ± 1.1	0.010*
Severity of ARDS			0.033*
Mild (n)	9	3	
Moderate (n)	20	12	
Severe (n)	15	24	
Biological indicators			
sRAGE (ng/L)	1246.0 ± 543.7	1628.4 ± 565.7	0.002*
esRAGE (ng/L)	338.1 ± 72.9	287.5 ± 70.3	0.002*

Data are expressed as number or the mean ± standard deviation. Analysis performed using Cox’s regression analysis.

*p < 0.05.

However, the survivors had a significantly lower Murray lung injury score and APACHE II and SOFA II scores (Table 1; all *p* < 0.05). The nonsurvivors had a significantly higher ELWI (*p* = 0.040), a prolonged length of stay in the ICU (*p* = 0.032), and a longer duration of mechanical ventilation (*p* = 0.021). According to the Berlin definition, 3 patients were classified as mild ARDS, and 12 and 24 were classified as moderate or severe ARDS, respectively. The survival was significantly higher in mild ARDS than in moderate or severe ARDS (*p* = 0.033) (Table 1).

The serum level of esRAGE was significantly higher in the survivor group than in the nonsurvivor group (*p* < 0.05), and the serum level of sRAGE was significantly higher in the nonsurvivor group (*p* < 0.05) (Table 1).

### Univariate and multivariate regression analyses of the clinical parameters for survival status in ARDS patients

Despite substantial differences in some clinical parameters between the survivors and nonsurvivors, it is unclear whether these differences are of clinical significance. To determine the potential prognostic factors of survival in ARDS patients, further univariate and multivariate regression analyses, which included the 9 variables relevant to the prognosis of disease progression mentioned above, were performed. Univariate analysis showed that serum levels of sRAGE and esRAGE, ELWI, MLIS, APACHE II score, severity of ARDS, and length of stay in the ICU were closely associated with ARDS (Table 2, univariate analysis). However, multivariable logistic regression analysis indicated that only the serum levels of sRAGE and esRAGE and ELWI were significant factors for prognosis in ARDS (Table 2, multivariate regression analysis).

**Table 2:** Contribution of various potential prognostic factors to survival in ARDS patients.

Variable	Univariate analysis				Multivariate analysis			
	OR	95% CI		*p* value	OR	95% CI		*p* value
Mechanical ventilation (hours)	1.004	1.000	1.008	0.053				
ICU length of stay (days)	1.099	1.006	1.200	0.036*	1.163	0.997	1.356	0.079
ELWI	1.171	1.004	1.364	0.044*	1.783	1.279	2.485	0.001*
SOFA II	1.179	1.005	1.383	0.043*	1.136	0.866	1.491	0.357
APACHE II	1.089	1.010	1.174	0.027*	1.134	0.989	1.301	0.072
MLIS	1.884	1.144	3.102	0.013*	1.778	0.855	3.697	0.123
Severity of ARDS	2.335	1.197	4.554	0.013*	2.373	0.841	6.695	0.102
sRAGE	1.001	1.000	1.002	0.005*	1.003	1.001	1.005	0.002*
esRAGE	0.990	0.983	0.997	0.004*	0.990	0.980	0.999	0.036*

*p < 0.05.

### The related parameters predicting 28-day mortality in ARDS patients

**Figure 2:**
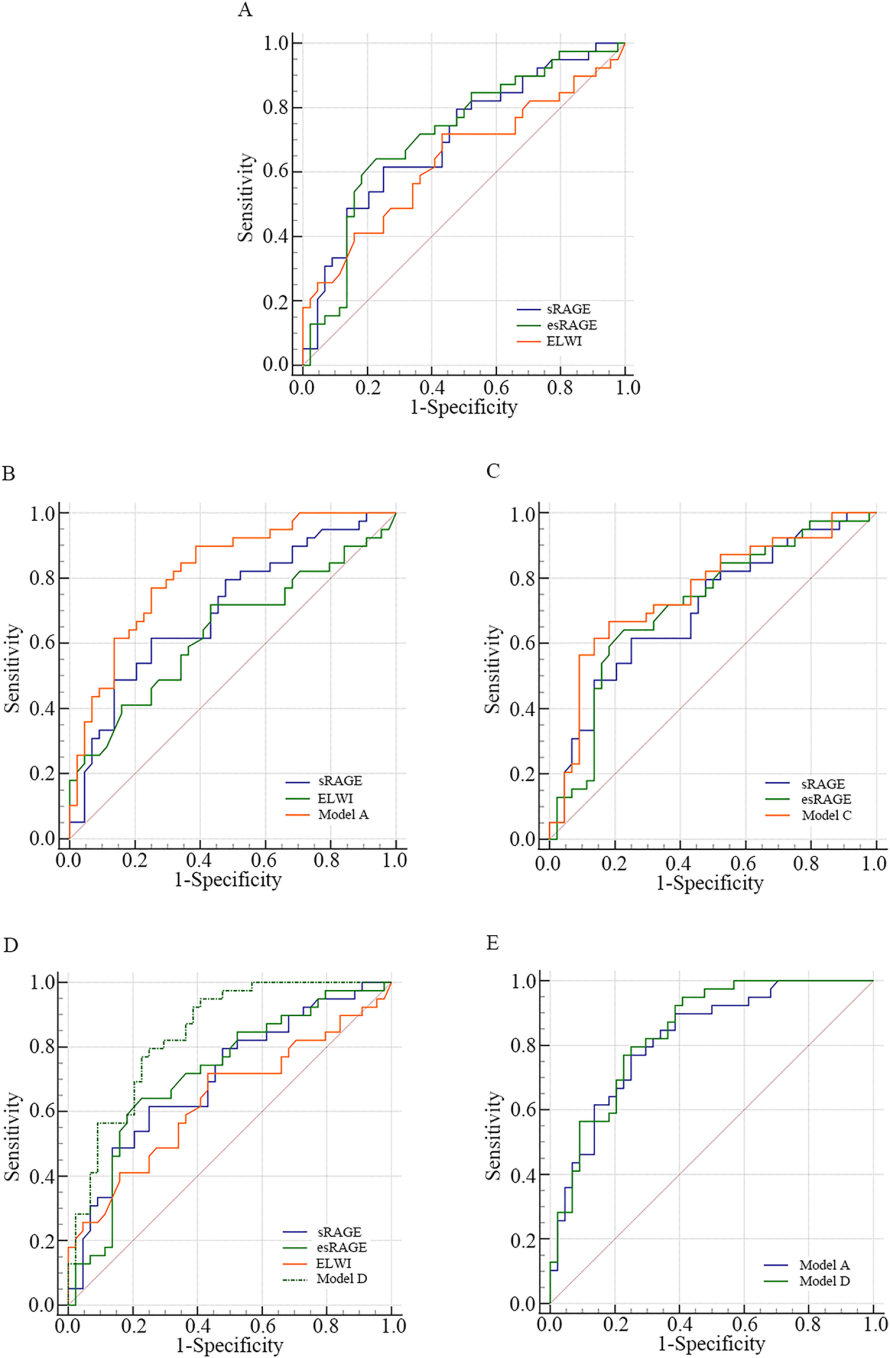
ROC curves were compared among the different models measuring the area under the curve (AUC).

To determine the independent predictors of 28-day mortality, we assessed the predictive validity of ELWI and the serum levels of sRAGE and esRAGE by receiver operator curves (ROC). ELWI and the serum levels of sRAGE and esRAGE were independent predictors of mortality, with AUC values of 0.637, 0.704, and 0.717, respectively (Table 3; Fig. 2A; all *p* < 0.05). There were no statistically significant differences in the AUC among the three indicators (Table 4; all *p* > 0.05).

**Table 3:** Prediction for the 28-day mortality of ARDS with the related parameters.

Variable	Cutoff	AUC	SE	95% CI		Youden index	Z value	*p* value
ELWI	12	0.637	0.063	0.515	0.760	0.286	2.192	0.028*
sRAGE (ng/L)	1518	0.704	0.058	0.565	0.804	0.365	3.535	0.004*
esRAGE (ng/L)	302	0.717	0.058	0.604	0.830	0.414	3.766	0.002*

Analysis performed using Receiver operator curve (ROC).

*p < 0.05.

**Table 4:** AUC of ELWI; sRAGE and or esRAGE in predcting the mortality in ARDS.

Variable	Difference	SE	95% CI		Z value	*p* value
ELWI versus sRAGE	0.067	0.106	− 0.140	0.274	0.632	0.528
ELWI versus esRAGE	0.080	0.084	− 0.084	0.244	0.960	0.337
sRAGE versus esRAGE	0.013	0.080	− 0.141	0.171	0.167	0.867

Analysis performed using Z-test.

*p < 0.05.

### Construction of the prognosis prediction model based on the pairwise combination strategy among ELWI, sRAGE, and esRAGE

Subsequently, we performed a pairwise combination strategy among ELWI, sRAGE, and esRAGE to predict 28-day mortality in ARDS patients. To determine the feasibility of the prediction model, the probability values of ELWI, sRAGE and esRAGE in different models were assessed by logistic regression analysis. In contrast with Model A (sRAGE + ELWI), Model C (sRAGE + esRAGE), and Model D (sRAGE + esRAGE + ELWI), Model B (esRAGE + ELWI) could not be developed into a valid linear regression model because the coefficient of ELWI did not show statistical significance (Table 5; *p* > 0.05), suggesting that Model B cannot be exploited to predict the 28-day mortality in ARDS patients (Table 5).

**Table 5:** The probability of different combination models in ARDS.

Variable	Coefficient	*p* value	Equation
Model A			
Constant	− 9.109	< 0.001*	Y = 0.427*ELWI + 0.003*sRAGE − 9.109
ELWI	0.427	< 0.001*	
sRAGE	0.003	< 0.001*	
Model B			
Constant	1.174	0.434	
ELWI	0.142	0.079	
esRAGE	− 0.010	0.006*	
Constant	1.036	0.421	
Model C			
sRAGE	0.001	0.008*	Y = 0.001*sRAGE-0.01*esRAGE
esRAGE	− 0.010	0.006*	
Model D			
Constant	− 6.057	0.016*	Y = 0.386*ELWI + 0.002sRAGE − 0.007esRAGE-6.057
sRAGE	0.002	< 0.001*	
esRAGE	− 0.007	0.004*	
ELWI	0.386	0.001*	

Analysis performed using logistic regression analysis. Model A: sRAGE + ELWI; Model B: esRAGE + ELWI; Model C: sRAGE + esRAGE; Model D: sRAGE + esRAGE + ELWI;

*p < 0.05.

According to Youden’s index^25^, the cut-off values of Model A and Model D were 0.401 (AUC 0.820, sensitivity 76.9%, specificity 75.0%) and 0.426 (AUC 0.841, sensitivity 79.5%, specificity 75.0%), respectively, in predicting the 28-day mortality of ARDS (Table 6). The predictive value of Model A or Model D was slightly better than that of ELWI, sRAGE level, or esRAGE level alone (Tables 6 and 7, Fig. 2B,D).

**Table 6:** Characteristics of different models for predicting the 28-day mortality of ARDS.

Variable	Cutoff	TPR	TNR	AUC	SE	95% CI	*p* value
Model A	0.401 (Y^1^)	0.769	0.750	0.820	0.046	0.731–0.910	< 0.001*
Model C	0.495 (Y^2^)	0.667	0.818	0.757	0.055	0.650–0.864	< 0.001*
Model D	0.426 (Y^3^)	0.795	0.750	0.841	0.043	0.757–0.924	< 0.001*

Analysis performed using Receiver operator curve (ROC). Y^1^ = 0.427*ELWI + 0.003*sRAGE − 9.109; Y^2^ = 0.001*sRAGE − 0.01*esRAGE; Y^3^ = 0.386*ELWI + 0.002sRAGE − 0.007esRAGE − 6.057;

*p < 0.05.

**Table 7:** AUC of differernt models in predcting the 28-day mortality of ARDS.

Variable	Difference	SE	95% CI	Z value	*p* value
Model A versus sRAGE	0.067	0.106	− 0.140–0.274	2.040	0.041*
Model A versus ELWI	0.183	0.062	− 0.061–0.305	2.938	0.003*
Model C versus sRAGE	0.053	0.048	− 0.040–0.146	1.116	0.265
Model C versus esRAGE	0.040	0.043	− 0.047–0.123	0.993	0.351
Model D versus sRAGE	0.137	0.058	0.024–0.250	2.382	0.017*
Model D versus esRAGE	0.124	0.053	0.020–0.227	2.339	0.019*
Model D versus ELWI	0.204	0.064	0.079–0.328	3.200	0.001*
Model A versus Model D	0.021	0.021	− 0.021–0.063	0.966	0.334

Analysis performed using Z-test.

*p < 0.05.

At a cut-off value of 0.495, the AUC of Model C was 0.757, with a sensitivity of 66.7% and a specificity of 81.8% (Table 6). The AUC of Model C was higher than that of the sRAGE level or esRAGE level alone, but with no statistically significant difference (Tables 6 and 7 and Fig. 2C), suggesting that Model C had a predictive value similar to that of the serum levels of sRAGE and esRAGE alone, which does not have an advantage in predicting 28-day mortality compared with Model A and Model D.

Meanwhile, there was no statistically significant difference in AUC between Model A and Model D (Table 7 and Fig. 2E). The above data indicate that Model A and Model D were more powerful in predicting the outcome of ARDS than the related parameters alone.

## Discussion

In this study, two biomarkers (namely, sRAGE and esRAGE) were measured in the blood to explain the associated clinical features of ARDS. The expression of esRAGE increased persistently in survivors of ARDS, and the serum sRAGE level increased persistently in nonsurvivors of ARDS. Multivariate logistic regression analysis showed that sRAGE/esRAGE levels and ELWI were independent risk factors for the prognosis of ARDS. Moreover, the sRAGE/esRAGE levels and ELWI alone also had a favourable predictive capability for 28-day mortality in patients with ARDS. Youden’s index analysis showed that Model A (sRAGE + ELWI) and Model D (sRAGE + esRAGE + ELWI) had a better predictive power for the prognosis of ARDS than the related parameters alone.

ARDS is a diffuse uncontrolled alveolar inflammatory disease, the hallmarks of which are alveolar damage, lung microvascular leakage, and overt noncardiogenic pulmonary oedema^1,26^. The inflammatory response plays an important role in the progression of ARDS, and it involves the infiltration of various immune cells, including neutrophils, macrophages, and lymphocytes, into the lung tissues and small airways^26^. RAGE, as a multiligand receptor of the immunoglobulin superfamily, is able to induce intracellular signalling pathways that regulate several inflammatory diseases by binding a diverse array of ligands, such as advanced glycation end products (AGEs), S100/calgranulins, HMGB1 proteins, amyloid-β peptides and the family of β-sheet fibrils^27^. For instance, inhibition of the HMGB1/RAGE axis by RAGE knockout reduced the activity of microglia-mediated neuroinflammation in a mouse model of stress-induced hypertension^28^. In limb ischaemia and reperfusion models, the inhibition of RAGE binding with HMGB1 by FPS-ZM1 significantly decreased the release of HIF-1a, NLRP3, Caspase-1, TNF-a, and IL-6 expression to mediate the inflammatory response^29^. Although serum sRAGE/esRAGE levels have been demonstrated to be involved in multiple inflammatory diseases, it is still unknown whether they participate in the ARDS-induced inflammatory response. This was the first study to evaluate sRAGE/esRAGE expression and its prognostic value in patients with ARDS, and we found that the plasma sRAGE level was significantly higher in the ARDS group than in the non-ARDS group, but the plasma esRAGE level was significantly lower in the ARDS group, indicating a positive correlation between the severity of ARDS and sRAGE expression, which may be reversed by esRAGE. The most reasonable explanation was probably that esRAGE, as a competitive inhibitor, bound with the same ligands and precluded cell-bound RAGE signalling, which attenuated the inflammatory-immune response. Therefore, the expression of serum esRAGE had organ-protecting and anti-inflammatory effects, and this finding has also been confirmed in the literature. For instance, the maternal serum esRAGE concentration and esRAGE/sRAGE ratio were significantly higher in patients with preeclampsia than in healthy pregnant controls^30^. The upregulation of esRAGE might also be part of the cell's antioxidative defences against plaque formation as a result of oxidative stress in the T2DM phenotype^31^. With the above consideration, we speculated that sRAGE expression increased as the disease worsened and that esRAGE expression increased as the disease improved, which was helpful for the early assessment of the prognosis in ARDS and might become an objective marker. In addition, multivariate analysis retained serum sRAGE/esRAGE levels as independent predictors of 28-day mortality in the ARDS patients in this study.

Numerous potential prognostic factors were associated with survival in patients with ARDS. Extravascular lung water (EVLW) is the generic name for the fluid within the lung but outside the vasculature, including extravasated plasma and intracellular water, which could be induced by elevated osmotic pressure and pulmonary vascular permeability. Kor et al.^32^ demonstrated that ELWI was a predictive marker for distinguishing ARDS-induced postoperative pulmonary oedema from cardiogenic pulmonary oedema, ultimately leading to impaired oxygenation and lung compliance. In our study, not only ELWI but also sRAGE and esRAGE had a close correlation with mortality in patients with ARDS by multivariate analysis. Furthermore, according to the statistically significant differences, ELWI, sRAGE, and esRAGE were chosen as independent predictors to establish the prediction model of 28-day mortality in ARDS patients.

Both scoring systems and real-time monitoring systems are highly sensitive but lack specificity for the progression of the disease. The biological indicators, although lacking sensitivity, had the advantage of being highly specific with the temporal requirements for protein synthesis. Theoretically, compared with any parameter alone, the pairwise combination strategy among ELWI, sRAGE, and esRAGE could significantly increase the sensitivity and specificity of predictive ability for 28-day mortality in ARDS patients. Nevertheless, Li et al.^33^ examined whether changes in the level of serum fibroblast growth Factor 21 (FGF21) and Sequential Organ Failure Assessment (SOFA) scores could predict the 28-day mortality of ICU patients with sepsis and ARDS, and no significant difference was observed between the FGF21 and SOFA scores. Therefore, these clinical indicators had considerable variability in predicting the disease status. Similarly, our data analysis revealed that no statistically significant changes were found in predicting the mortality of ARDS in analyses of ELWI versus sRAGE, ELWI versus esRAGE, or sRAGE versus esRAGE, suggesting that ELWI, sRAGE, and esRAGE possessed a similar predictive value for disease progression in patients with ARDS.

Nevertheless, there were also relatively large heterogeneities in ELWI, sRAGE, and esRAGE, which might cause offsets and increase the risk of misjudgement in disease prediction. Thus, the combined forecasting model was established to improve the predictive power^34^. Interestingly, except for Model A and Model D, other combinations failed to increase the AUC for the prediction of mortality in ARDS. These negative results might be explained by the limited samples and medical intervention before ICU admission, which could bias the evaluation results. Nevertheless, we could still conclude that ELWI, sRAGE, and esRAGE were predictors of mortality in ARDS, and the combination of sRAGE and ELWI was able to increase the power of predicting the prognosis of ARDS. To date, the relationship between esRAGE level and the mortality of ARDS has not been reported elsewhere in the literature.

Moreover, the plasma sRAGE/esRAGE levels and ELWI, based on the statistical analysis results, may be used to assess prognosis in patients with ARDS alone. With the popularization of the invasive monitoring technique PiCCO, ELWI has the advantage of low cost and easy acquisition. However, during the data collection by the thermodilution technique, the accuracy of ELWI is affected by the skill level of the operator^35,36^, which indicates that ELWI is not a pure objective indicator. In contrast to ELWI, the plasma sRAGE/esRAGE level measurement has the potential to be an objective prognostic tool because it is an easy, objective, economical approach with minimal tissue trauma. In addition, Model A (sRAGE + ELWI) and Model D (sRAGE + esRAGE + ELWI) showed stronger predictive power than ELWI, sRAGE and esRAGE alone. From the data analysis perspective, Model A did not have a statistically superior predictive advantage over Model D. However, according to the reasonable allocation of medical resources and optimal socioeconomic benefits, Model A (sRAGE + ELWI) may serve as the preferred regimen for predicting the prognosis of ARDS.

Although strict inclusion and exclusion criteria were used, the present study still had several limitations: (1) this was a single-centre study, and the sample size was limited. An expanded sample size is needed in future studies. (2) The timing of the sample collection was limited, and the lack of dynamic inspection might also be partly attributable to detection bias. In addition, we failed to observe changes in bronchoalveolar lavage fluid (BALF) biomarkers, which might induce an underestimation of the value of these biomarkers. (3) Owing to the relatively small volume of blood collected and limited funds, it was impossible to detect and research all biomarkers for ARDS (such as biomarkers related to epithelial injury, endothelial injury, and other inflammatory factors). (4) The number of cases with mild ARDS was relatively small. In this regard, the assessment of mild ARDS might be biased to a certain extent. Accordingly, the generalization of our results to other ARDS patients requires some caution.

## Conclusions

Our study demonstrated that the admission level of sRAGE was higher in nonsurviving ARDS patients, while the serum esRAGE level showed the opposite trend, and the use of the plasma sRAGE/esRAGE levels was helpful for the assessment of the prognosis in ARDS and might be an objective marker. Model A (sRAGE + ELWI) and Model D (sRAGE + esRAGE + ELWI) had stronger predictive power for predicting the 28-day mortality of ARDS. Our results are conducive to the early distinction of critical ARDS patients and the early intervention and management of ARDS.

## Data Availability

The datasets used and/or analysed during the current study are available from the corresponding author upon reasonable request.

## Abbreviations

ARDS: Acute respiratory distress syndrome
RAGE: Receptor for advanced glycation end products
sRAGE: Elevated plasma levels of cell surface RAGE
esRAGE: Endogenous secretory RAGE
ELWI: Extravascular lung water index
SOFA: Sequential organ failure assessment
APACHE II: Acute physiology and chronic health evaluation
MLIS: Murray lung injury score
PiCCO: Pulse indicator continuous cardiac output
ROC: Receiver operating characteristic curve
